# Supplementary material for: Clinical Distribution and Drug Resistance of Pseudomonas aeruginosa in Guangzhou, China from 2017 to 2021
Source: J Clin Med. 2023 Feb 2;12(3):1189. doi: 10.3390/jcm12031189 (PMC9917919; doi:10.3390/jcm12031189)
Supplement: Supplementary file 1 [file jcm-12-01189-s001.zip › jcm-2052750-supplementary.pdf]

Supplementary Data:

Table S1. Treatment course of patients with XDR-PA infection.

Year	Patient	AMK	GEN	TOB	ATM	CAZ	FEP	CIP	LEV	IMP	MEM	PIP	TZP	Diagnosis of disease	Empirical treatment	Antibiotic therapy	Prognosis
2017	1	R	R	R	S	R	R	R	R	R	R	R	R	Chronic obstructive pulmonary disease, Stage 2 hypertension		IMP	Improved but not cured
	2	R	R	R	R	R	R	R	R	R	R	R	R	Pneumonia, type 2 diabetes mellitus, Stage 2 hypertension, hypothyroidism, Sequelae of cerebral infarction.	CSTS, IMP	IMP, TZP	Not getting better
	3	R	R	R	R	R	R	R	R	R	R	R	R	Tracheal stenosis, Tracheostomy status, Intracranial injury.		TZP, AZM	Improved but not cured
	4	R	R	R	R	R	R	R	R	R	R	R	R	Lung infection, Respiratory failure, Nasopharyngeal carcinoma (post-radiotherapy), Urinary tract infection.	CSSS, MEM	CSSS, MEM	Improved but not cured
2018	5	R	R	R	R	R	R	R	R	R	R	R	R	Lung infection, Pleural effusion, Heart disease Stage 3 hypertension, Type 2 diabetes mellitus, hypoproteinemia, parkinson's disease.		Imipenem/Cilastatin Sodium	Death
	6	R	R	R	R	R	R	R	R	R	R	R	R	Pneumonia, Stage 2 hypertension.	CSSS	LEV	Not cured
	7	S	R	R	R	R	R	R	R	R	R	R	R	Intracerebral hemorrhage, Basal ganglia hemorrhage, vascular dementia, Stage 3 hypertension, Paroxysmal atrial fibrillation, left anterior branch block, cardiac function grade II, Cholecystitis, Pancreatic cyst, Chronic cystitis,		TZP, VAN, MEM	Death

													Eczema			
2019	8	R	R	R	R	R	R	R	R	R	R	R	R	Cardiogenic shock, Septic shock, lung infection, Stage 3 hypertension, Type 2 diabetes is associated with complications.		MEM, VAN, Polymyxin B	Death
	9	R	R	R	R	R	R	R	R	R	R	R	R	Fever, Exfoliative dermatitis, Renal failure, Cirrhosis, Type 2 diabetes.	CSSS, MEM	CSSS, VAN	Not cured
	10	R	R	R	R	R	R	R	R	R	R	R	R	Parkinson's disease, Osteoporosis, Intertrochanteric fracture, Essential hypertension, Breast cancer; Cholecystitis.	CSSS,	Imipenem/Cilastatin Sodium, VAN, LEV , MEM	Not cured
	11	R	R	R	R	R	R	R	R	R	R	R	R	Mass in esophagus, Stage 3 hypertension, Multiple diabetic complications, lacunar infarction, Chronic obstructive pulmonary disease.		CSSS, VAN, Imipenem/Cilastatin Sodium , TZP, MEM	Not cured
	12	S	R	R	R	R	R	R	R	R	R	R	R	Chronic obstructive pulmonary disease, Atelectasis, Stage 2 hypertension, Parkinson's disease, Arrhythmia.		Compound Glycyrrhiza	Cured
	13	R	R	R	R	R	R	R	R	R	R	R	R	Severe pneumonia, Viral encephalitis, Pleural effusion, Emphysema, Asthma complications, Lacunar infarction, Chronic obstructive pulmonary disease.	TZP	LEV, MEM	Death
2020	14	R	R	R	R	S	R	R	R	R	R	R	R	Myelodysplastic syndrome, Severe pneumonia, Acute respiratory distress syndrome, septic shock, Acute renal failure, post-cholecystectomy.	CZA, Colistin Sulphate	Colistin Sulphate, CAZ	Not cured
	15	R	R	R	R	R	R	R	R	R	R	R	R	Respiratory failure, lung infection, Anticardiolipin antibody syndrome, Stage 3 hypertension,	Imipenem/Cilastatin	Polymyxin B	Not cured

														Moderate anemia, Stage 3 chronic kidney disease	Sodium		
	16	S	R	R	R	R	R	R	R	R	R	R	R	Acute peritonitis, Acute appendicitis with perforation, lung infection.	MEM	MEM, LEV AMK, Imipenem/Cilastatin Sodium	Not cured
	17	R	R	R	R	R	R	R	R	R	R	R	R	Pneumonia, Pubic fracture, lumbar degenerative disease, Rib fracture, Osteoporosis, Chronic heart failure, Arrhythmia type of coronary heart disease, Valvular heart disease, Atrial fibrillation, Stage 2 hypertension, Anemia, Pressuresore.		Meropenem, piperacillin/tazobactam, vancomycin, CZA	Death
	18	R	R	R	R	R	R	R	R	R	R	R	R	Septic shock, Myelosuppression, Myelodysplastic syndrome, Type 2 diabetes, Pleural effusion, Emphysema, Coronary atherosclerosis, Atherosclerosis of the aorta.		Imipenem/Cilastatin Sodium, VAN, Polymyxin B	Death
2021	19	R	R	R	I	R	R	R	R	R	R	R	R	Pneumonia.		TZP, Polymyxin B	Cured
	20	R	R	R	R	R	R	R	R	R	R	R	R	Aortic arch aneurysm, Coronary artery bypass grafting, Lung infection, Pulmonary hypertension, Right heart failure, Kidney failure, Paroxysmal atrial fibrillation, Paroxysmal ventricular tachycardia, Multiple serous cavity effusion, Emphysema, Hepatic cyst, Unilateral inguinal hernia without obstruction or gangrene.	MEM, polymyxin B, colistin Sulphate, LEV	CZA, Colistin sulfate, MEM, Polymyxin B	Not cured
	21	R	R	R	R	R	R	R	R	R	R	R	I	Sepsis, Septic shock, Severe pneumonia, Necrosis of the ileum , Abdominal wall soft tissue infection, Fungal pneumonia, Myocardial infarction,	LEV, Polymyxin B	Imipenem/Cilastatin Sodium, MEM, VAN	Death

														Cardiogenic shock, Acute renal failure, liver failure, Multiple organ failure, Pleural effusion.			
	22	R	R	R	R	R	R	R	R	R	R	R	R	Heart failure, Atrial fibrillation, Pleural effusion, Stage 1 hypertension, Type 2 diabetes, Carotid thrombosis cerebral infarction, Foot soft tissue infection, Valvular heart disease.		LEV, TZP, CZA, cefotaxime/sulbactam	Death
	23	R	R	R	R	R	R	R	R	R	R	R	R	Parkinson's syndrome, Essential hypertension, Chronic bronchitis, Bladder disease.	MEM	TZP, CSSS, CAZ, MEM, VAN, Polymyxin B	Not cured
	24	R	R	R	R	R	R	R	S	R	R	R	R	Renal arteriolar sclerosis, Stage 4 chronic kidney disease, Sequelae of cerebral infarction, Hypertensive retinopathy, Type 2 diabetes.		CSSS	Cured
	25	R	R	R	R	R	R	R	R	R	R	R	R	Liver cancer, Cerebral herniation, Pneumonia, Respiratory failure, Pleural effusion, Hepatic cyst, Cholecystitis without Gallstone.		MEM	Death
	26	R	R	R	R	R	R	R	R	R	R	R	R	Esophageal malignancy, Pneumonia, Atelectasis, Abdominal aortic aneurysm.	Imipenem/Cilastatin Sodium, LEV	MEM, Colistin Sulphate	Cured

Abbreviation: AMK:amikacin ; GEN: gentamicin; TOB: tobramycin; ATM: aztreonam; CAZ: ceftazidime; FEP: cefepime, CIP: ciprofloxacin; LEV :Levofloxacin; IMP: Imipenem, MEM: meropenem; PIP: Piperacillin ;TZP; Piperacillin-tazobactam; CSTS: Cefoperazone Sodium/Tazobactam Sodium for Injection; CSSS: Cefoperazone Sodium and Sulbactam Sodium for

Injection; VAN:Vancomycin; AZM: Azithromycin; CZA:Ceftazidime/Avibactam; R: resistance;S: susceptible.
